# Anhydroexfoliamycin, a *Streptomyces* Secondary Metabolite, Mitigates Microglia-Driven Inflammation

**DOI:** 10.1021/acschemneuro.1c00033

**Published:** 2021-06-10

**Authors:** Sandra Gegunde, Amparo Alfonso, Rebeca Alvariño, Nadia Pérez-Fuentes, Luis M. Botana

**Affiliations:** † Departamento de Farmacología, Facultad de Veterinaria, Universidad de Santiago de Compostela, 27002 Lugo, Spain; ‡ Grupo Investigación Biosdiscovery, IDIS, 15706 Santiago de Compostela, Spain

**Keywords:** Microglia, neuron, inflammation, neuroprotection, antioxidant, *Streptomyces*

## Abstract

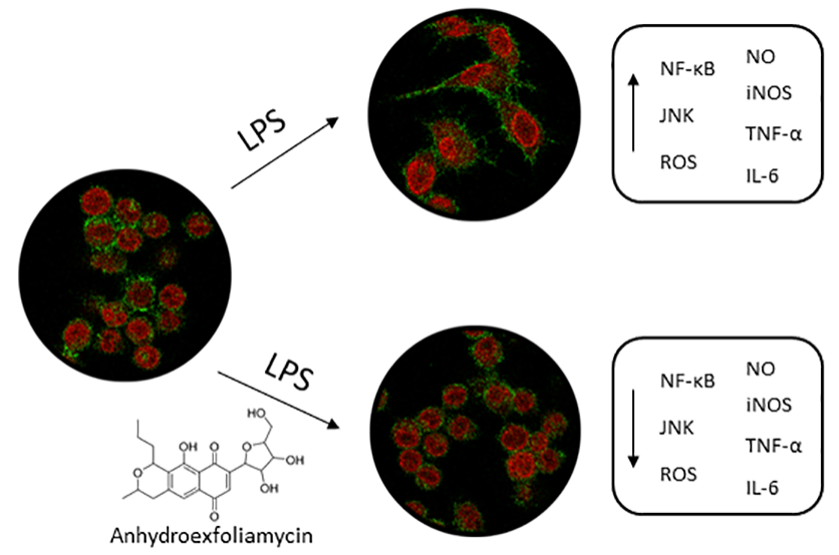

Anhydroexfoliamycin, a secondary metabolite from *Streptomyces*, has shown antioxidant properties in primary cortical neurons reducing neurodegenerative hallmarks diseases, both *in vitro* and *in vivo* models. Activated microglia, in the central nervous system, plays a crucial role in neuroinflammation and is associated with neurodegeneration. Therefore, the aim of the present study was to determine the anti-inflammatory and antioxidant potential of the anhydroexfoliamycin over microglia BV2 cells. Neuroinflammation was simulated by incubation of microglia cells in the presence of lipopolysaccharide to activate proinflammatory transduction pathways. Moreover, a coculture of neuron SH-SY5Y and microglia BV2 cells was used to evaluate the neuroprotective properties of the *Streptomyces* metabolite. When microglia cells were preincubated with anhydroexfoliamycin, proinflammatory pathways, such as the translocation of the nuclear factor κB, the phosphorylation of c-Jun N-terminal kinase, and the inducible nitric oxide synthase expression, were inhibited. In addition, intracellular reactive oxygen species generation and the liberation of nitric oxide, interleukin 6, and tumor necrosis factor α were also decreased. Besides, the *Streptomyces*-derived compound showed antioxidant properties promoting the translocation of the factor erythroid 2-related factor 2 and protecting the SH-SY5Y cells from the neurotoxic mediators released by activated microglia. The effects of this compound were at the same level as the immunosuppressive drug cyclosporine A. Therefore, these results indicate that anhydroexfoliamycin is a promising tool to control microglia-driven inflammation with therapeutic potential in neuroinflammation.

## 1 Introduction

Microglia, a macrophage-like population, is crucial in the central nervous system (CNS) as host defense, neuronal homeostasis, and tissue repair.^1^ In a healthy brain, resident microglia are mainly responsible for innate immunity and become activated in response to stressful stimuli such as injury, damaged cells, and infections.^2^ Depending on the stimulus that causes microglia activation, microglia can adopt polarized phenotypes, ranging from proinflammatory M1 phenotype (classical activation) to M2 immunosuppressive phenotype (alternative activation/acquired deactivation).^3^ Proinflammatory microglia activation is associated with proinflammatory cytokines release, such as proinflammatory interleukins (IL) and tumor necrosis factor α (TNF-α), as well as with the production of nitric oxide (NO) and reactive oxygen species (ROS) increase.^4^ Initially, this activation represents the first line of defense in the brain, where microglia are the main component of neuroinflammation in the CNS. Nevertheless, when neuroinflammation becomes chronic, activated microglia are detrimental to neuronal cells, participating in the progression of several neurodegenerative disorders, namely, Alzheimer’s disease, Parkinson’s disease, and amyotrophic lateral sclerosis.^3,5,6^ Meanwhile, the M2 phenotype is characterized by its neuroprotective functions that are associated with increased IL-4, neurotrophic factors, and proteases release, among others. Furthermore, M2 microglia activity leads to the removal of tissue debris as well as to the reduction of inflammation without causing neuronal cells’ dysfunction and death.^7,8^ Therefore, in neuroinflammation-mediated neurodegenerative disease, changing the M1 phenotype to the beneficial M2 by drug treatment would be an interesting therapeutic target.

Several factors can participate in the microglia activation, such as environmental factors, age, peptides or proteins (observed in neurodegenerative disorders, like Alzheimer’s disease or Parkinson’s disease), and accumulation of toxic metabolites, contributing to the neuroinflammatory pathway.^9^ Microglia express some immune receptors, including toll-like receptors (TLRs). The presence of proinflammatory stimuli increases the expression of TLRs that are responsible for pathogen-associated molecular pattern recognition.^10^ TLRs are a type I transmembrane protein responsible for immune response; nevertheless, an improper TLR response leads to acute and chronic inflammation.^11^ Lipopolysaccharide (LPS), an endotoxin of the outer membrane of the Gram-negative bacteria, through TLR4 signaling induces an inflammatory response in the microglia. The binding of LPS to TLR4 results in the activation of several transduction pathways, such as transcription factor nuclear factor (NF) κB and mitogen-activated kinases (MAPK), which enhance the production of proinflammatory cytokines and inducible nitric oxide synthase (iNOS), among others, resulting in neuroinflammation.^10,11^ Hence, inhibiting NF-κB or MAPK pathway activation is gaining relevance as a therapeutic approach to alleviate the progression of neuroinflammation.

**Figure 1:**
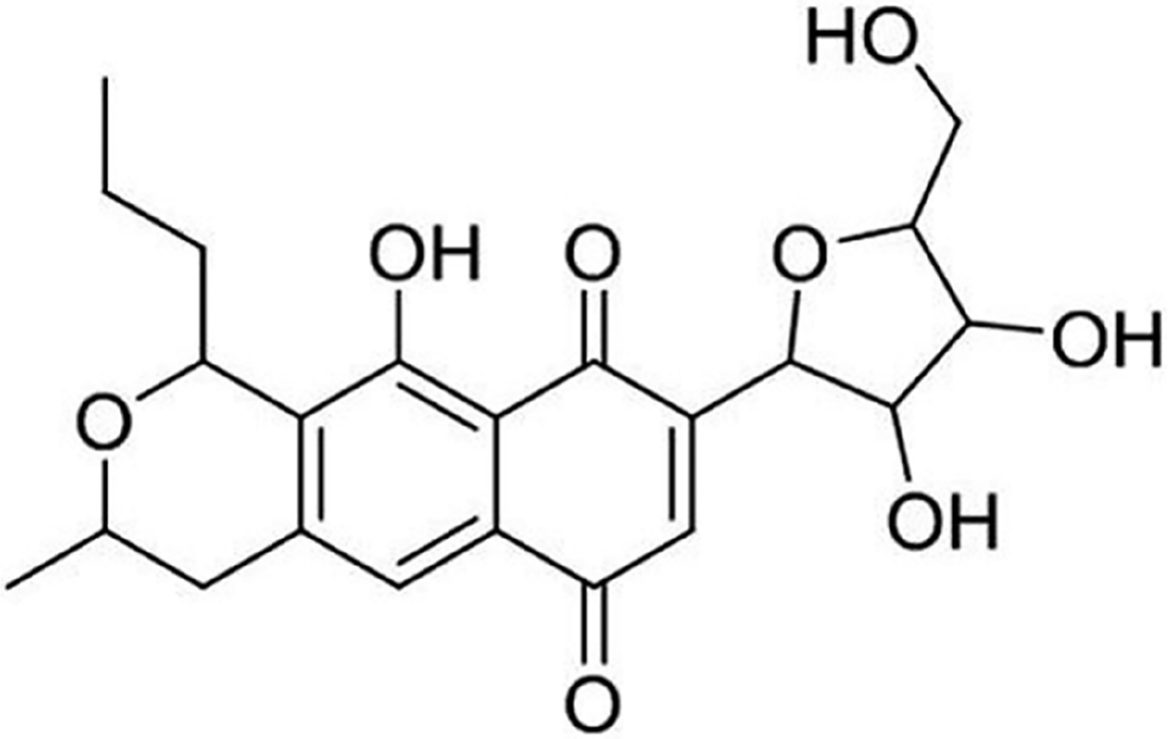
Structure of anhydroexfoliamycin.

*Streptomyces*, a Gram-positive filamentous bacteria group, is the largest and most studied genus of *Actinobacteria*.^12^*Streptomyces* species are prolific producers of a significant number of metabolites with diverse properties.^13^ Moreover, this genus is the source of several clinical compounds with immunosuppressive, antitumor, anthelmintic, antifungal, and antibiotic activities, among others.^14−16^ Furthermore, some *Streptomyces* secondary metabolites have shown neuroprotection against neurodegenerative disorders.^17,18^ Anhydroexfoliamycin (AE), a *Streptomyces* secondary metabolite, has shown antioxidant properties in primary cortical neurons through the improvement of mitochondrial dysfunction, reduction of ROS generation, and enhancement of antioxidant enzymes (Figure 1).^19^ Moreover, this metabolite reduced hallmarks of Alzheimer’s disease, such as Aβ and tau proteins, in *in vitro* and *in vivo* models.^20^ Considering that neuroinflammation can be triggered by oxidative stress and is widely associated with the pathogenesis of Alzheimer’s disease, the potential protective effects of AE were tested against the inflammation LPS-induced in microglia cells.

## 2 Results and Discussion

The present study describes the effects of the *Streptomyces* secondary metabolite, AE, over an *in vitro* inflammatory model. In this cellular model, LPS was used to activate microglia BV2 cells, re-creating neuroinflammatory conditions. In consequence, NF-κB and JNK MAPK pathways were activated, and the release of proinflammatory cytokines, including IL-6 and TNF-α, as well as the production of NO and ROS, was increased. Moreover, this is a suitable cell model of microglia inflammation since 90% of the genes induced by LPS in BV2 microglia cells are also induced in primary microglia.^21^

*Streptomyces*, from the genus *Actinobacteria*, has contributed to the development of two-thirds of the currently used antibiotics and so secondary metabolites of great interest. Moreover, natural products may serve as the basis to supply lead compounds with therapeutic potential in pathologies with an inflammatory origin. Numerous natural products have been widely used in the treatment of inflammatory-related diseases in clinical settings, including triptolide, resveratrol, and silymarin.^22,23^*Streptomyces* secondary metabolites, from species in soil and marine ecosystems, are also a rich source of new compounds for drug discovery.^24^ The hyperarid Atacama Desert (Chile) is one source of secondary metabolites from *Streptomyces* sp., including the AE used in the present study.^25,26^ AE has shown interesting antioxidant properties. We have tested the *Streptomyces* derivative on primary cortical neurons and showed neuroprotection against H_2_O_2_ damage, maintaining the mitochondrial membrane potential, reducing ROS, and enhancing antioxidant enzyme levels through nuclear factor erythroid 2-related factor 2 (Nrf2) translocation to the nucleus.^19^ Moreover, in an *in vitro* model of Alzheimer’s disease, some kinases, including JNK, that control tau phosphorylation and Aβ42 levels were reduced by AE.^20^ In addition, we have also tested AE in 3xTg-AD mice, a transgenic model of AD that also develops neuroinflammation.^27^ In these *in vivo* experiments, the AE protects against AD pathology since it was able to ameliorate major hallmarks of AD through kinases modulation and reducing of Aβ and tau proteins.^20^ The promising results of this metabolite in neurodegenerative disorders led us to check its effect over activated microglia. Since several intracellular processes and cells are altered in neuroinflammation-related diseases, compounds able to target major hallmarks of the disease and to reduce neuroinflammation would be welcome.^28^ Also, this compound has never been tested in inflammatory models.

**Figure 2:**
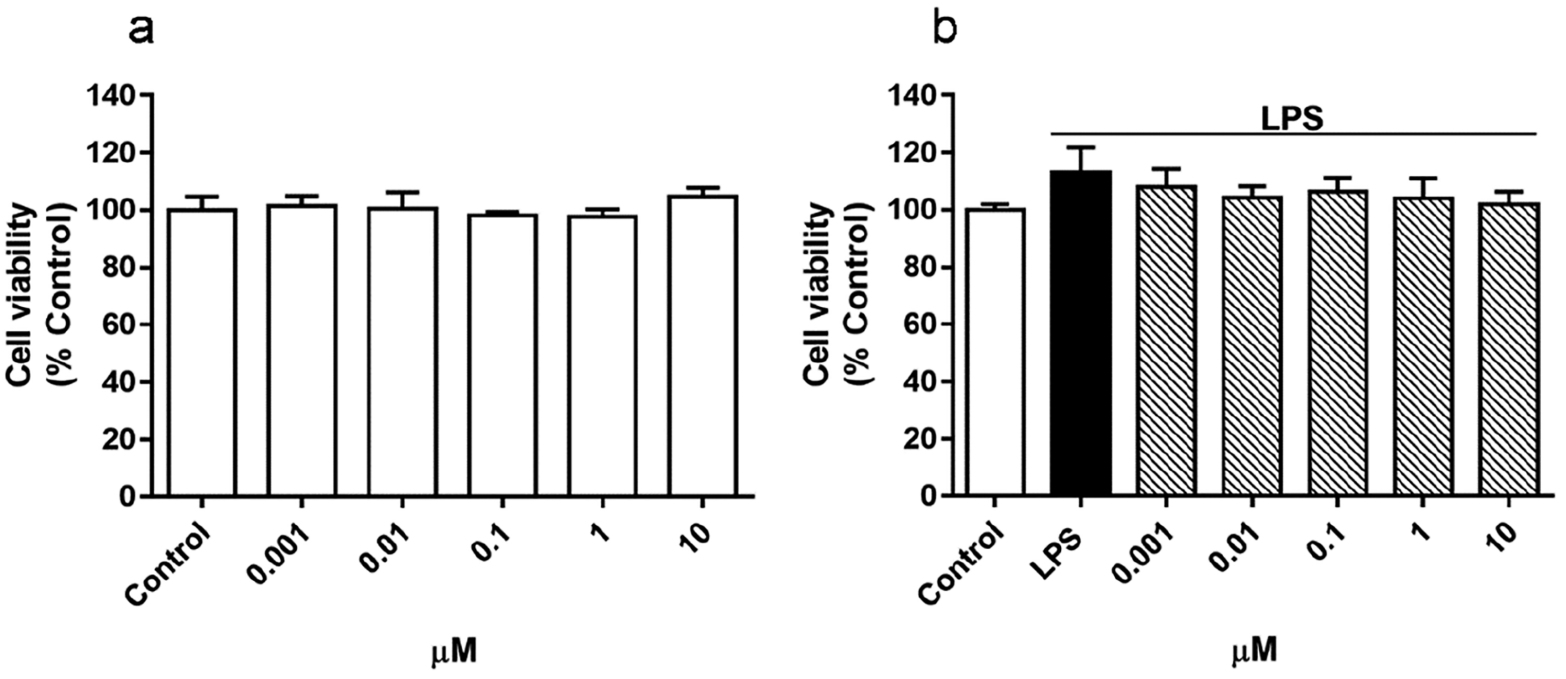
Effect of AE on cell viability in microglia BV2 cells. Cells were treated with AE at different concentrations (0.001, 0.01, 0.1, 1, and 10 μM) for 24 h (a) or with different AE concentrations (0.001, 0.01, 0.1, 1, and 10 μM) plus LPS (500 ng/mL) (b). Cell viability was measured by MTT assay. Data are represented as a percentage, being the result of mean absorbance ± SEM of a minimum of *N* = 3 independent experiments performed in triplicate. The values are shown as the difference between control cells versus cells treated by ANOVA statistical analysis followed by post hoc Dunnet’s *t*-test. AE: anhydroexfoliamycin. LPS: lipopolysaccharide.

For this purpose, AE was tested in an inflammatory cellular model to evaluate its effects over inflammation since microglia-mediated neuroinflammation is implicated in the development of neurodegenerative diseases.^3^ Therefore, microglia BV2 cells were used, as they are frequently employed to test compounds for anti-inflammatory properties. Furthermore, LPS stimulates the activation and therefore the inflammatory response of microglia.^29,30^ First, the effect of AE over cell viability was assessed to determine if AE would affect microglial cells. As Figure 2a shows, no cytotoxic effects were found by the *Streptomyces* derivative at any concentration tested (0.001–10 μM). From previous work, 500 ng/mL of LPS was used to activate BV2 cells, since this concentration does not modify cell viability but can stimulate inflammatory conditions.^31^ Then, the effect of AE (0.001–10 μM) over cell viability in the presence of 500 ng/mL of LPS was also determined. As Figure 2b shows, after 24 h of incubation with AE and LPS, no modifications over cell survival were found. Therefore, since the concentrations tested did not show cytotoxicity, for neuroinflammation experiments, the most effective concentration of AE (0.1 μM) in previous experiments was chosen.^19,20^

**Figure 3:**
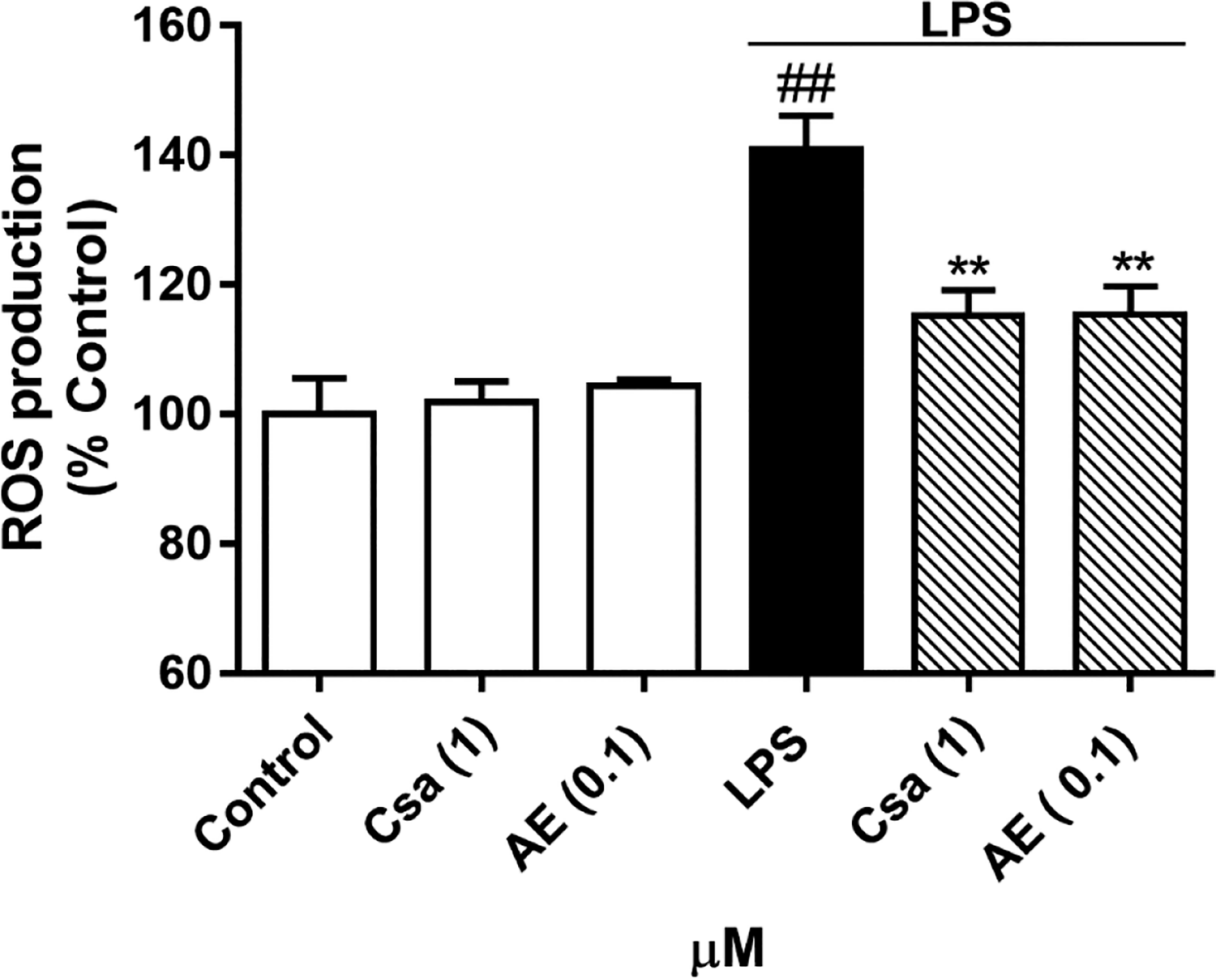
Effect of AE on intracellular ROS production in LPS-stimulated microglia BV2 cells. Cells were pretreated with AE at 0.1 μM for 1 h and then were stimulated with LPS (500 ng/mL) for 24 h. CsA was used as a control of anti-inflammatory effects (1 μM). ROS levels were measured with DCFH-DA. Data are represented as a percentage, being the result of mean fluorescence intensity ± SEM of a minimum of *N* = 3 independent experiments performed in triplicate. The values are shown as the difference between cells treated with LPS alone versus cells treated with compounds in the presence of LPS by ANOVA statistical analysis followed by post hoc Dunnet’s *t*-test: **p < 0.01, or cells treated with LPS versus control cells; ^##^*p* < 0.01. AE: anhydroexfoliamycin. CsA: cyclosporine A. LPS: lipopolysaccharide.

Inflammation and microglia activation promote oxidative stress and DNA damage leading to ROS overproduction. In addition to ROS causing direct cytotoxicity, it also triggers the activation of proinflammatory pathways. Mitochondria are the main source of ROS, and the excessive production of ROS by the electron transport chain or impairment of antioxidant defense causes mitochondrial dysfunction and the initiation of cell death and neurodegeneration.^32^ Therefore, the effect of AE over ROS in BV2 cells activated by LPS was measured. Cyclosporine A (CsA) was used as a control of anti-inflammatory effects since it is a well-known anti-inflammatory and immunosuppressive drug.^31^ As Figure 3 shows, when cells were treated with LPS, the ROS production was 40% increased (*p* < 0.01) compared with untreated cells. When BV2 was pretreated 1 h with AE before the LPS stimulation, ROS levels were decreased in a dose-dependent fashion. In this sense, the pretreatment with AE reduced the ROS production stimulated by LPS at the same level as CsA control (*p* < 0.01). Both compounds restored ROS levels up to control values. Furthermore, several studies have described that the LPS-induced inflammatory response in microglia is directly associated with ROS overproduction and, by direct reduction of ROS release, the inflammatory response is inhibited.^33,34^ Therefore, the inhibition of ROS generation by the *Streptomyces* secondary metabolite may lead to inflammatory response reduction.

**Figure 4:**
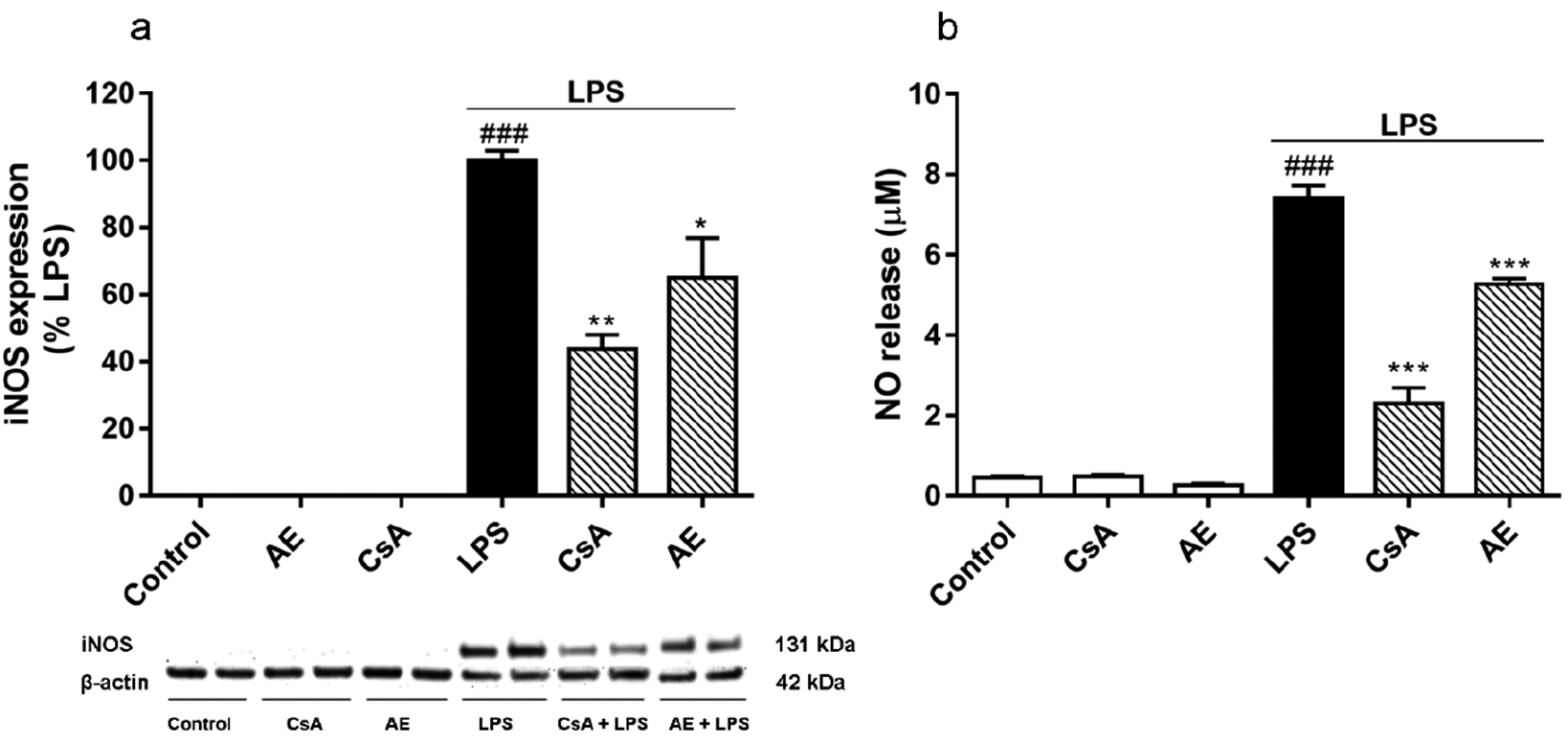
Effect of AE on iNOS expression and the generation of NO in LPS-stimulated microglia BV2 cells. Cells were pretreated with AE 0.1 μM for 1 h and then were stimulated with LPS (500 ng/mL) for 24 h. CsA was used as a control of anti-inflammatory effects (1 μM). iNOS expression was measured in cytosolic lysates by Western blot (a). NO was measured by Griess reagent (b). Data are represented as a percentage, being the result of the mean ± SEM of a minimum of *N* = 3 independent experiments performed in duplicate. The values are shown as the difference between cells treated with LPS alone versus cells treated with compounds in the presence of LPS by ANOVA statistical analysis followed by post hoc Dunnet’s *t*-test: *p < 0.05, **p < 0.01, ***p < 0.001, or cells treated with LPS versus control cells; ^###^*p* < 0.001. AE: anhydroexfoliamycin. CsA: cyclosporine A. LPS: lipopolysaccharide.

In the CNS, NO acts as a neurotransmitter and cellular messenger. Nevertheless, under pathological conditions, microglia cells express large amounts of iNOS, which catalyzes the production of NO. In these conditions, NO becomes a toxic free radical exacerbating tissue inflammation and induces cell apoptosis, oxidative stress, and neuronal impairment.^30,35^ Furthermore, high TNF-α levels are critical in the pathogenesis of inflammation and enhance the production of toxic substances, including NO.^8^ Then, the next step was to investigate the effect of AE in the expression of iNOS and the NO release. iNOS expression was measured in the cytosolic lysates of BV2 cells after 24 h of treatments. As Figure 4a shows, as expected, iNOS expression was not observed in the cytosol of LPS-unstimulated cells. Nevertheless, when BV2 cells were LPS-activated, iNOS expression was detected. Moreover, iNOS levels were decreased, almost 40%, by the *Streptomyces*-derived compound or CsA (*p* < 0.05). Hence, to check if iNOS reduction affects the NO release, nitrite (a marker of NO production) concentration was measured in cell supernatants. In the medium of cells treated with LPS the NO concentration was 7.41 ± 0.31 μM. Meanwhile, in the medium of the untreated cell it was 0.46 ± 0.03 μM (Figure 4b). As happens with iNOS expression, the NO release was significantly decreased, 30%, by AE pretreatment (*p* < 0.001). Also, CsA notably reduced the NO liberation (*p* < 0.001). Therefore, AE significantly reduced both iNOS expression and NO release, suggesting its anti-inflammatory and neuroprotective potential. In this sense, it has been demonstrated that silenced iNOS expression has neuroprotective effects in an animal model of Parkinson’s disease and microglial activation is reduced.^36^

**Figure 5:**
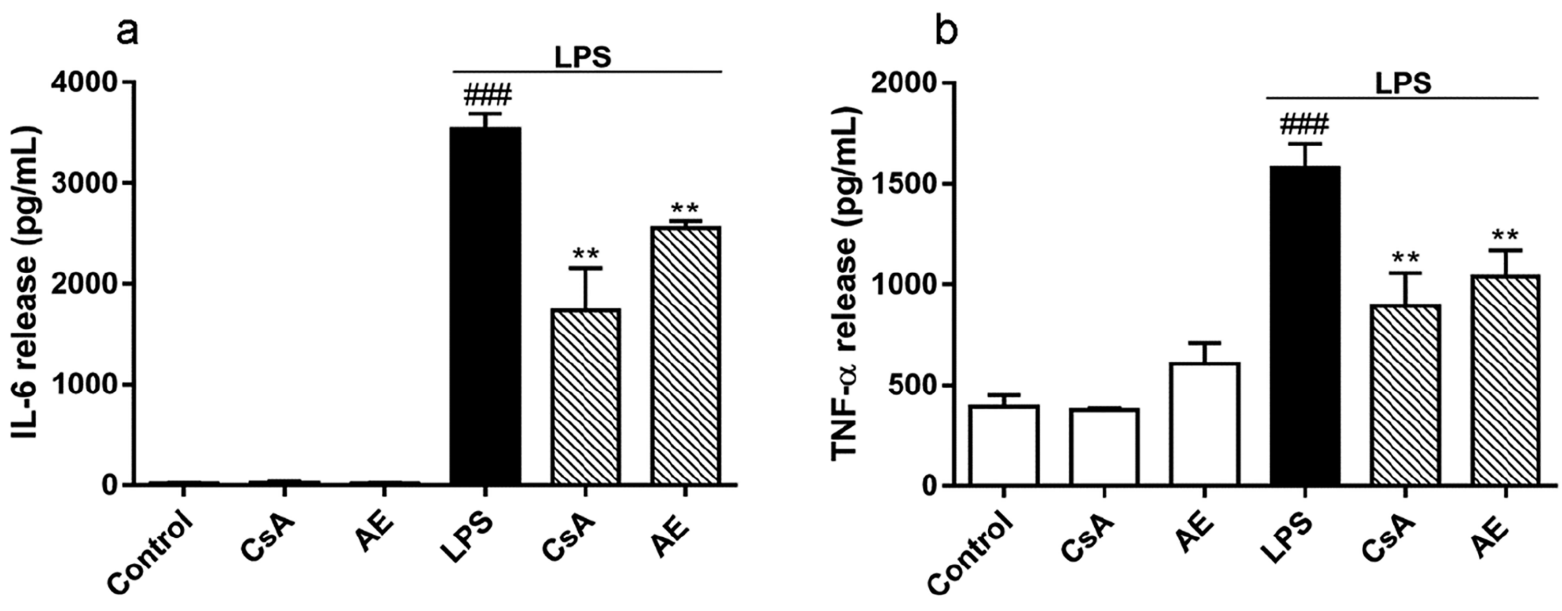
Effect of AE on the release of IL-6 and TNF-α in LPS-stimulated microglia BV2 cells. Cells were pretreated with AE at 0.1 μM for 1 h and then were stimulated with LPS (500 ng/mL) for 24 h. CsA was used as a control of anti-inflammatory effects (1 μM). IL-6 (a) and TNF-α (b) levels were measured by ELISA. Data are represented as a percentage, being the result of the mean ± SEM of a minimum of *N* = 3 independent experiments performed in duplicate. The values are shown as the difference between cells treated with LPS alone versus cells treated with compounds in the presence of LPS by ANOVA statistical analysis followed by post hoc Dunnet’s *t*-test: **p < 0.01, or cells treated with LPS versus control cells; ^###^*p* < 0.001. AE: anhydroexfoliamycin. CsA: cyclosporine A. LPS: lipopolysaccharide.

LPS acts as a ligand for TLR4 and activates the inflammatory cascade, inducing downstream signal pathways such as NF-κB.^10^ The transcription factor NF-κB regulates multiple processes, namely, inflammation, apoptosis, immune response, and cell survival, among others. Nevertheless, NF-κB activation in glial cells causes the activation of M1 phenotypic microglia resulting in neuroinflammation, participating in the pathogenesis of neurodegenerative diseases.^11^ The NF-κB activation depends on its translocation from the cytosol to the nucleus. In normal conditions, NF-κB is retained in the cytosol bound to the inhibitory protein IκB. The inflammatory stimuli, in this case, LPS, promote the phosphorylation and proteasomal degradation of IκB and result in the nuclear translocation of NF-κB p65 subunit, producing proinflammatory cytokines and mediators, including TNF-α and IL-6.^9^ Moreover, the levels of TNF-α and IL-6 are increased in neurodegenerative diseases and microglia injury, and their concentrations were also measured in the cellular media of LPS-stimulated BV2.^37^ Hence, as Figure 5a shows, in the medium of unstimulated cells, IL-6 was barely detected. Nevertheless, high IL-6 amounts were observed in LPS-stimulated BV2 cells medium (3533.66 ± 158.28 pg/mL). IL-6 release by LPS-stimulated cells was significantly inhibited, 30%, by the pretreatments with the *Streptomyces* derivative compound or CsA (*p* < 0.01). In the case of TNF-α, Figure 5b, its release in activated microglia was also increased (1579.31 ± 119.22 pg/mL) more than 3 times, compared with control cells (*p* < 0.001). Furthermore, in the presence of AE, TNF-α concentration was reduced (1036.82 ± 76.86 pg/mL) in the medium, at the same level as CsA. Thus, AE reduces the release of proinflammatory cytokines by activated microglia.

**Figure 6:**
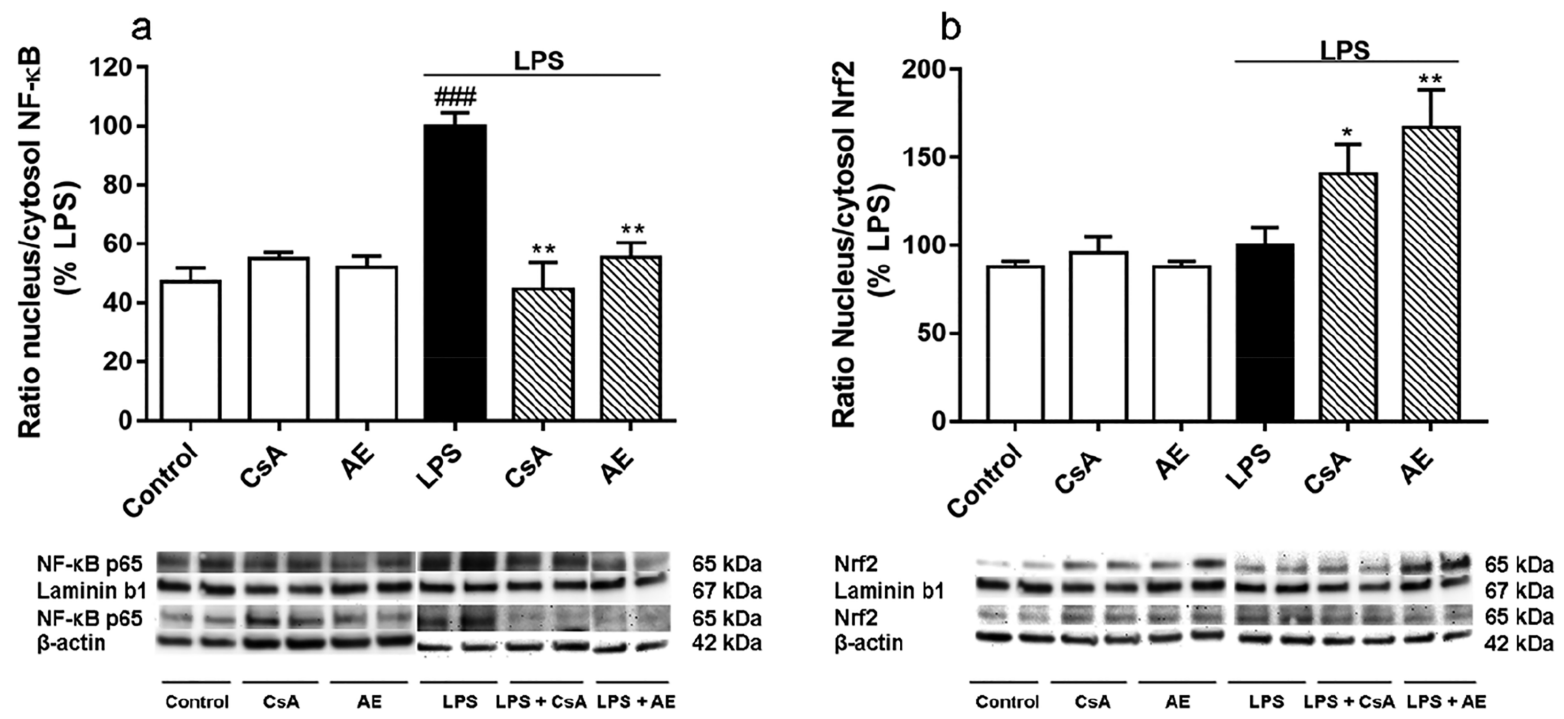
Effect of AE on the expression of NF-κB and Nrf2 in LPS-stimulated microglia BV2 cells. Cells were pretreated with AE at 0.1 μM for 1 h and then were stimulated with LPS (500 ng/mL) for 24 h. CsA was used as a control of anti-inflammatory effects (1 μM). The NF-κB p65 subunit expression was measured in cytosolic and nucleus lysates by Western blot and represented as nucleus/cytosol ratio (a). Nrf2 expression was measured in cytosolic and nucleus lysates by Western blot and represented as nucleus/cytosol ratio (b). Data are represented as a percentage, being the result of the mean absorbance ± SEM of a minimum of *N* = 3 independent experiments performed in triplicate. The values are shown as the difference between cells treated with LPS alone versus cells treated with compounds in the presence of LPS by ANOVA statistical analysis followed by post hoc Dunnet’s *t*-test: *p < 0.05, **p < 0.01, or cells treated with LPS versus control cells; ^###^*p* < 0.001. AE: anhydroexfoliamycin. CsA: cyclosporine A. LPS: lipopolysaccharide.

The transcription factors NF-κB and Nrf2 in microglia are modulators of their proinflammatory and antioxidant responses, respectively.^38^ So, given the results above, the translocation from cytosol to the nucleus of NF-κB and Nrf2 was assessed by Western blot. Then NF-κB p65 and Nrf2 were measured in the cytosol and nucleus and their translocation ratios were calculated. As Figure 6a shows, the NF-κB p65 translocation was doubled when BV2 was activated with LPS (*p* < 0.001). The NF-κB p65 translocation was significantly inhibited with the pretreatments with AE or CsA (*p* < 0.001). Thus, the inhibition of NF-κB could foster the phenotypic shift of M1 to M2. Moreover, recent studies have shown that the regulation of the microglial phenotype through NF-κB downregulation has neuroprotective effects as inflammation is reduced.^39,40^ Besides, the proinflammatory mediators NF-κB stimulated, such as TNF-α and IL-6, cause blood–brain barriers (BBB) disruption, allowing lymphocytes to enter the brain exacerbating CNS inflammatory response.^37^ Also, elevated levels of TNF-α and IL-6 in the brain have been associated with neuronal damage and neuroinflammation. Besides, TNF-α mediates the apoptosis of neurons.^9^ Thus, targeting both TNF-α and IL-6 prevents not only CNS inflammation but also neuronal death. From our results, it could be hypothesized that the inhibition of TNF-α and IL-6 by AE could be related to the NF-κB blockage. Therefore, these results suggest that mediating NF-κB translocation by AE may reduce the expression of proinflammatory mediators and cytokines, alleviating the activation of BV2.

When Nrf2 expression was measured, no significant differences were detected between LPS-stimulated BV2 cells and controls (Figure 6b). However, the metabolite AE significantly enhances the translocation of Nrf2 in activated microglial cells (*p* < 0.01) to a larger extent than CsA control (*p* < 0.05). Hence, the *Streptomyces* derivative inhibits NF-κB p65 translocation and meanwhile promotes Nrf2 translocation. Nrf2 is a redox-sensitive transcription factor, which activates antioxidant and cytoprotective genes and enzymes upon oxidative conditions. Also, Nrf2 modulates microglia phenotype, from the proinflammatory phenotype in favor of the anti-inflammatory. In the present study, AE acted as an Nrf2 inductor and reduced ROS levels in activated microglia. These results agree with previous studies; the AE has shown interesting neuroprotective and antioxidant activities, acting on mitochondria. ROS productions were reduced by this compound in primary cortical neurons; moreover, it improved antioxidant enzyme levels and the Nrf2 translocation.^19^ However, despite several antioxidant compounds that have been effective in alleviating neurodegenerative disorders in *in vitro* experiments, in most *in vivo* studies they did not show neuroprotective or anti-inflammatory effects.^41^ This inefficacy in animal models is attributed to the instability of compounds or due to the difficulty of the compounds reaching brain cells, among others.^32^ The beneficial effects shown by AE in rodents against Alzheimer’s disease hallmarks indicate that this compound crosses the BBB, predicting a possible favorable *in vivo* role of this compound.^20^

**Figure 7:**
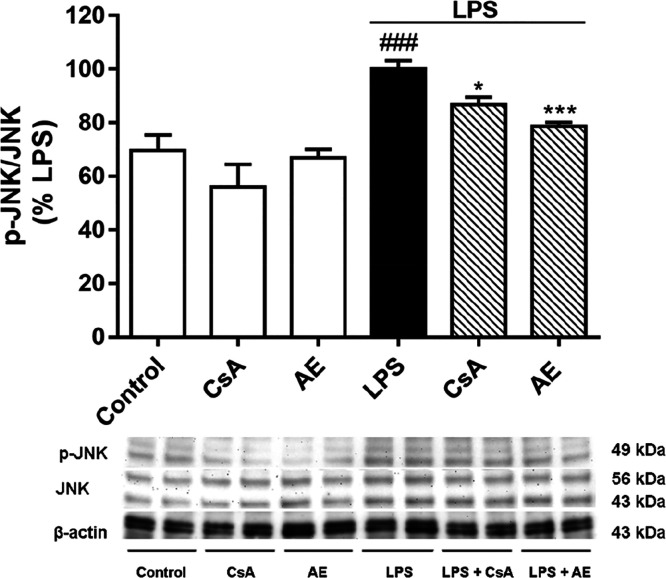
Effect of AE on the expression of JNK in LPS-stimulated microglia BV2 cells. Cells were pretreated with AE at 0.1 μM for 1 h and then were stimulated with LPS (500 ng/mL) for 24 h. CsA was used as a control of anti-inflammatory effects (1 μM). The total-JNK expression was measured in cytosolic and phosphorylated-JNK in nucleus lysates by Western blot and represented as nucleus/cytosol ratio. Data are represented as a percentage, being the result of the mean absorbance ± SEM of a minimum of *N* = 3 independent experiments performed in triplicate. The values are shown as the difference between cells treated with LPS alone versus cells treated with compounds in the presence of LPS by ANOVA statistical analysis followed by post hoc Dunnet’s *t*-test: *p < 0.05, ***p < 0.001, or cells treated with LPS versus control cells; ^###^*p* < 0.001. AE: anhydroexfoliamycin. CsA: cyclosporine A. LPS: lipopolysaccharide.

In addition to NF-κB pathway, MAPK signaling pathway activation is required for microglia-mediated CNS inflammation. Upon inflammatory stimuli or stressors and proinflammatory mediators such as TNF-α, among others, the MAPK signaling pathway is activated, including extracellular signal-regulated kinase (ERK) 1/2, c-Jun N-terminal kinase (JNK), and p38 pathway.^42^ Between them, JNK plays a key role in microglia activation and is responsible for tau phosphorylation in Alzheimer’s disease.^18^ Therefore, the effect of AE over the JNK MAPK signaling pathway was also tested. As shown in Figure 7, when BV2 cells were LPS-stimulated, the levels of phosphorylated JNK were increased. When cells were pretreated with AE, the phosphorylation of JNK was significantly suppressed, more than 20% (*p* < 0.001). In the case of CsA pretreatment, the JNK phosphorylation was 15% reduced. In this sense, these results suggest that JNK is an important molecular target of this compound. Moreover, JNK phosphorylation inhibition prevents tau phosphorylation and synaptic dysfunction. Furthermore, the inhibition of the JNK pathway helps to prevent the inflammatory response induced by overactivated microglia.^43^ Therefore, AE could be modulating the inflammatory cascade by JNK inhibition. However, more studies in other MAPK pathways (ERK 1/2 and p38) will be interesting.

**Figure 8:**
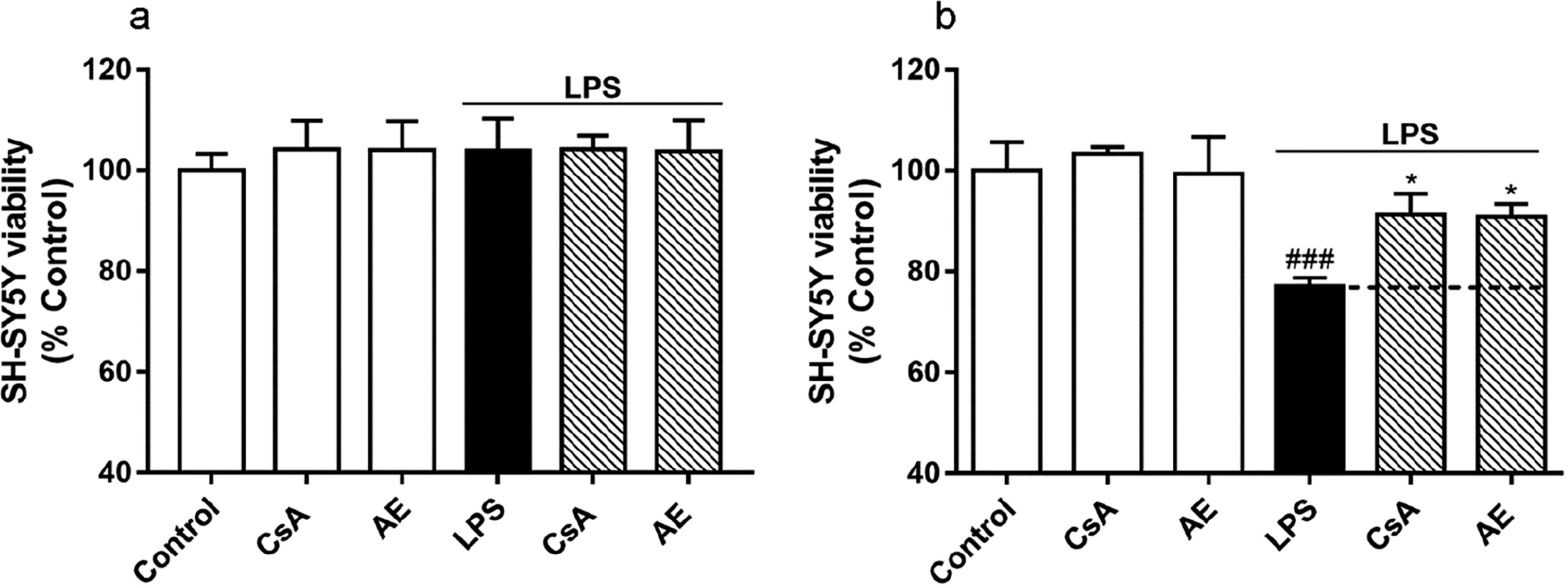
Effect of AE on SH-SY5Y survival microglia-neuronal coculture system. SH-SY5Y cells were pretreated with AE (0.1 μM) for 1 h and then were stimulated with LPS (500 ng/mL) for 24 h (a). SH-SY5Y cells were cultured with LPS-stimulated microglia BV2 cells with pretreatment of AE (0.1 μM) for 24 h (b). CsA was used as a control of anti-inflammatory effects (1 μM). MTT assay was performed to determine SH-SY5Y cell viability. Data are represented as a percentage, being the result of the mean absorbance ± SEM of a minimum of *N* = 3 independent experiments performed in triplicate. The values are shown as the difference between cells treated with LPS alone versus cells treated with compounds in the presence of LPS by ANOVA statistical analysis followed by post hoc Dunnet’s *t*-test: *p < 0.05, or cells treated with LPS versus control cells; ^###^*p* < 0.001. AE: anhydroexfoliamycin. CsA: cyclosporine A. LPS: lipopolysaccharide.

The release of proinflammatory factors such as cytokines, NO, and ROS by activated microglia is detrimental to neurons. For this reason, a coculture system with BV2 microglia and SH-SY5Y neuronal cells was used.^44^ With this system, the effects of toxicants released by microglia on neuronal cell viability can be checked. Thus, the anti-inflammatory effects of AE over activated microglia and how this affects neuronal viability were tested in the coculture system. Neuronal cells were grown at the bottom of the 24-well plate and microglial cells in inserts. Through the semipermeable membrane of the insets, the microglia and neuron cells share the environment, avoiding direct contact between them. First, the effect of AE or CsA plus LPS over neuronal cell viability in the absence of BV2 was tested. As Figure 8a shows, neither compound alone nor in the presence of LPS affects the viability of the SH-SY5Y cells. Then, the viability of SH-SY5Y in the presence of activated BV2 cells was determined. For this purpose, BV2 cells were placed in the insets of the coculture system and were pretreated with AE or CsA before the stimulation with LPS. As Figure 8b shows, the cell viability of neurons was 25% decreased (*p* < 0.001) when BV2 cells were activated by LPS. Nevertheless, the pretreatment with *Streptomyces* derivative or CsA significantly protected SH-SY5Y cells from the neurotoxic factors released by LPS-activated microglia, restoring cell viability almost to control cell values (*p* < 0.05). Therefore, AE protects the neurons against the damage caused by cytokines, NO, and ROS released by activated microglia.

**Figure 9:**
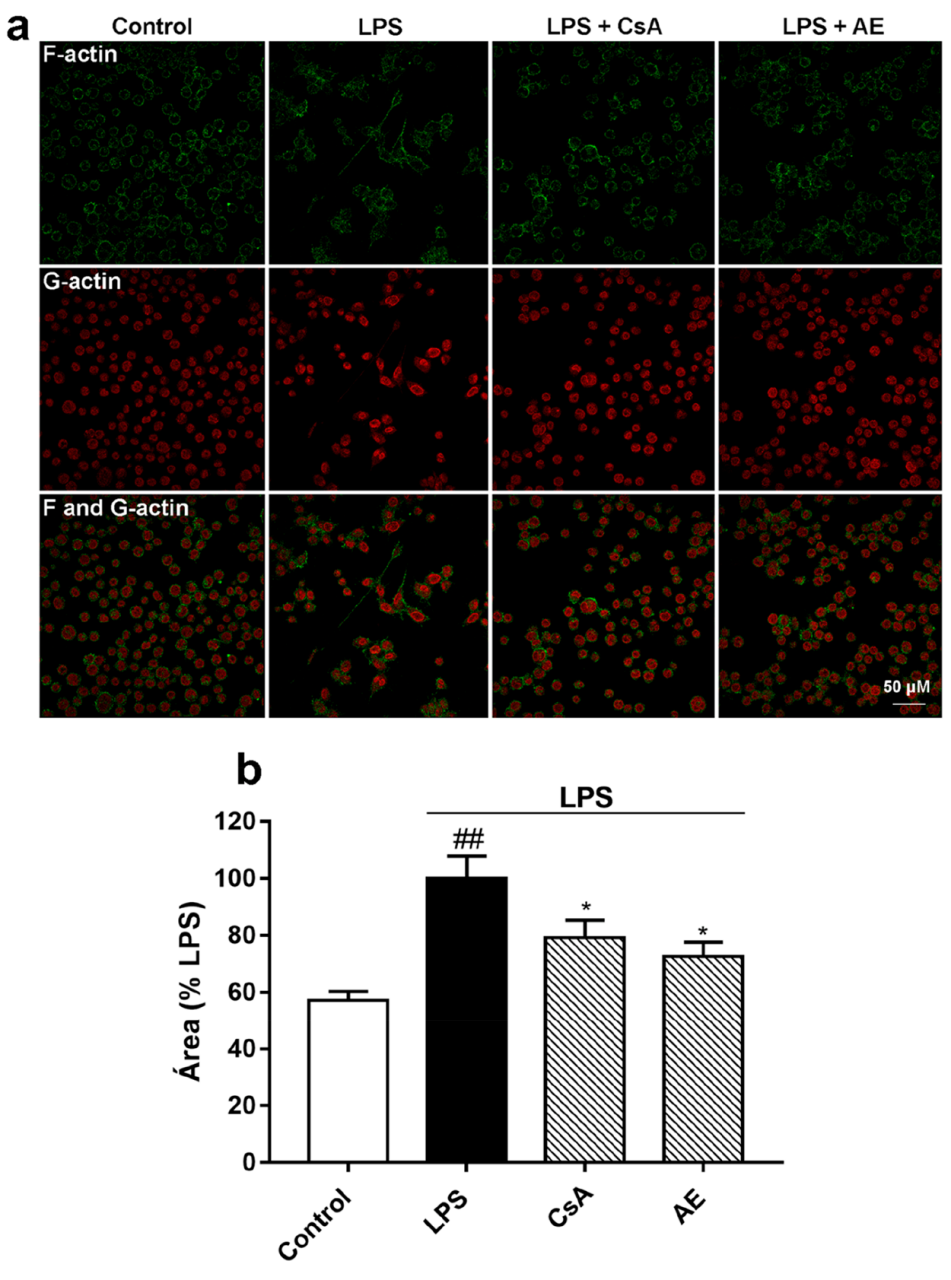
Effect of AE on BV2 cells actin cytoskeleton and morphology. Cells were pretreated with AE at 0.1 μM for 1 h and then were stimulated with LPS (500 ng/mL) for 24 h. CsA was used as a control of anti-inflammatory effects (1 μM). Confocal imaging of F-actin (green) and G-actin (red) double-staining of BV2 microglia cells (a). Images are representative of three independent experiments. Scale bar = 50 μM. BV2 cells area (b). Data are represented as a percentage, being the result of the mean ± SEM of a minimum of *N* = 3 independent experiments performed in duplicate. The values are shown as the difference between cells treated with LPS alone versus cells treated with compounds in the presence of LPS by ANOVA statistical analysis followed by post hoc Dunnet’s *t*-test: *p < 0.05, or cells treated with LPS versus control cells; ^##^*p* < 0.01. AE: anhydroexfoliamycin. CsA: cyclosporine A. LPS: lipopolysaccharide.

When microglia are activated, in addition to releasing proinflammatory mediators, their morphology is also altered.^45,46^ So it was checked if LPS causes morphological and/or cytoskeletal variations in BV2 cells. For this purpose, BV2 cells were grown in coverslips, followed by fixation and labeling with Oregon Green 514 phalloidin (green), a marker for F-actin, and with Texas Red DNase I (red), a marker for G-actin. As Figure 9a shows, unstimulated microglia cells were uniform in size and form with a round cytoplasm. When cells are treated with LPS, the size and cell form vary. The cytoplasm space was expanded with the formation of filopodia. Then, whether the *Streptomyces* derivative compound affects the morphological changes produced by LPS was checked. In the presence of EA, the cytoplasmic size and shape of the cells revert to those of the unstimulated cells, at the same level as CsA. The area of BV2 cells in the above conditions was also calculated. The area of LPS-stimulated cells was increased by almost 50% when compared with untreated cells (Figure 9b, *p* < 0.01). Moreover, with AE pretreatment the cell area is 30% decreased and 20% in the case of CsA pretreatments.

As above-described, after stimulation, microglia can switch its phenotype between proinflammatory M1 and immunosuppressive M2.^3^ In this sense, the present results suggest that the natural product AE could change the polarization of the microglia to the immunosuppressive phenotype since the natural compound promotes the nuclear translocation of Nrf2 rather than NF-κB. In addition, morphological changes and an increment in the cell area were observed in LPS-stimulated BV2 cells, which is a sign of M1 polarization.^46,47^ The ability of AE to minimize these morphological changes, in addition to the anti-inflammatory effects shown in this study, suggests that this compound reverses the phenotype M1 toward the anti-inflammatory M2. These data are consistent with current research, demonstrating that blueberry extracts shift the phenotype of LPS-stimulated BV2 cells for the immunosuppressive M2.^46^ Moreover, recent studies have outlined that the induction of M2 cell activation is a promising target in the context of neurodegenerative diseases.^39^ Moreover, from our results in the microglia and neuron coculture, AE is not neurotoxic. Inhibiting proinflammatory mediators released by activated microglia, AE can prevent neuronal cell death caused by microglia cell-driven neuroinflammation. Some *Streptomyces*-derived compounds have shown similar anti-inflammatory effects, as in the case of violacin A.^48^ These data confirm the anti-inflammatory functions of AE.

Finally, all experiments were compared with the well-known immunosuppressive CsA. It is widely used to reduce transplant rejection. Moreover, CsA has shown neuroprotective activities by preserving normal mitochondrial function.^49^ Nevertheless, its use in neurodegeneration is limited by its side effects and its high hepatic and nephrotoxicity.^50^ The present study, despite using AE at a lower dose than CsA, has shown similar or stronger anti-inflammatory effects compared with CsA.

## 3 Conclusions

In the present study, we showed that AE inhibits the inflammatory response in activated microglia. Moreover, it is the first evidence that AE inhibits LPS-induced NF-κB and JNK pathways activation and their subsequent inflammatory cascade, including the release of proinflammatory and neurotoxic mediators that are detrimental to neurons and can lead to neuron death. These data, together with the promising results shown in primary neurons and in *in vitro* and *in vivo* models of Alzheimer’s disease, suggest that AE acts on several neuroinflammation targets.^19,20^ Considering that neuroinflammation and degenerative diseases are multifactorial, this multitarget compound is a potential candidate in these pathologies’ treatment since it acted in the main hallmarks of the diseases. However, further studies would be necessary to better understand its action mechanism.

## 4 Materials and Methods

### 4.1 Chemicals and Solutions

The natural compound AE (Figure 1) was kindly donated by Dr. Marcel Jaspars from the Marine Biodiscovery Centre (Department of Chemistry, University of Aberdeen, Scotland, U.K.). This compound was isolated as described before.^19,20^ The stock solution of the compound was done in dimethylsulfoxide (DMSO).

Microglia BV2 cell line was obtained from InterLab Cell Line Collection (ICLC), number ATL03001 (Genova, Italy). The human neuroblastoma SH-SY5Y cell line was purchased from American Type Culture Collection (ATCC), number CRL2266. Dulbecco’s modified Eagle medium: Mix F-12 nutrient (DMEN/F-12), Roswell Park Memorial Institute Medium (RPMI), penicillin–streptomycin (10000 U/mL), trypsin/EDTA (0.05%), Glutamax, SuperSignal West Pico, SuperSignal West Femto, 7′,2′-dichlorofluorescein diacetate (DCFH-DA), tetramethylrhodamine methyl ester (TMRM), Griess reagent kit for nitrite quantitation, and anti-actin monoclonal antibody (catalog no. ACTN05 (C4), lot no. SK2474691D) were obtained from Thermo Fisher Scientific (Madrid, Spain). Magnetic bead panel Milliplex map kit (no. MHSTCMAG-70k), Millicell hanging cell culture insert (0.4 μm), polyvinylidene difluoride (PVDF) membrane, anti-NF-E2-related factor 2 antibody (catalog no. ABE413, lot no. 3035180), and anti-NF-κB p65 antibody (catalog no. ABE347, lot no. 2897243) were purchased from Merk (Madrid, Spain). Anti-lamin B1 antibody (catalog no. ab16048, lot no. GR3188002-1) and anti-iNOS antibody (catalog no. ab178945, lot no. GR324713-8) were bought in Abcam (Madrid, Spain). Anti-p-JNK/SAPK (pT183/pY185) (catalog no. 612540) and anti-JNK/SAPK1 (catalog no. 610627) were purchased from BD Biosciences. Polyacrylamide gels and molecular weight marker Precision Plus Protein Standards Kaleidoscope were obtained from Bio-Rad (Barcelona, Spain). Protease inhibitor Complete tablets and phosphatase inhibitor cocktail tablets were from Roche (Spain). 3-(4,5-Dimethylthiazol-2-yl)-2,5-diphenyltetrazolium bromide (MTT), sodium dodecyl sulfate (SDS), lipopolysaccharide, saponin from quillaja bark, bovine serum albumin (BSA), and the rest of the reagents used were purchased from Sigma-Aldrich (Madrid, Spain).

### 4.2 Cell Culture

Microglia BV2 cells were cultured in Roswell Park Memorial Institute Medium (RPMI) plus 10% fetal bovine serum (FBS), penicillin (100 U/mL), and 100 μg/mL streptomycin at 37 °C in a humidified atmosphere of 5% CO_2_ and 95% air. Cells were dissociated twice a week using 0.05% trypsin/EDTA (1×).

Neuroblastoma SH-SY5Y cells were maintained in Dulbecco’s modified Eagle Mmedium: Nutrient Mix F-12 (DMEN/F-12) supplemented with 10% FBS, 1% Glutamax, penicillin (100 U/mL), and 100 μg/mL streptomycin at 37 °C in a humidified atmosphere of 5% CO_2_ and 95% air. Cells were dissociated weekly using 0.05% trypsin/EDTA (1×).

### 4.3 Cell Viability Assay

Cell viability was assessed using the MTT assay, as previously described.^51^ Briefly, microglia BV2 cells were seeded in 96-well plates (at a density of 4 × 10^4^ cells per well) and exposed to different compound concentration (0.001, 0.01, 0.1, 1, and 10 μM) for 24 h or were pretreated with AE (0.001, 0.01, 0.1, 1, and 10 μM) 1 h before the stimulation with LPS (500 ng/mL) for 24 h. After incubation, cells were rinsed and incubated with MTT (500 μg/mL) diluted in a saline buffer for 1 h at 37 °C. Then, MTT excess was washed, cells were disaggregated with 5% SDS, and the absorbance of colored formazan salt was obtaining using a spectrophotometer plate reader (595 nm). Saponin was used as a cellular death control, and its absorbance was substrate from the other data.

### 4.4 Measurement of Intracellular ROS Production

Intracellular ROS levels were measured using DCFH-DA, as previously described.^52^ In brief, BV2 cells were treated with AE (0.1 μM) or CsA (1 μM) in the presence or absence of LPS (500 ng/mL). After 24 h, cells were rinsed twice with saline buffer and incubated 1 h at 37 °C with 20 μM DCFH-DA. During this time, DCFH-DA enters the cell and is deacetylated by cellular esterases, which is oxidized by ROS in 7′,2′-dichlorofluorescein (DCF). DCF is fluorescent and is detected on a spectrophotometer plate reader (495 nm excitation and 527 nm emission).

### 4.5 NO Release Determination

The release of NO to the culture medium by BV2 cells was established by measuring nitrite formed by the oxidation of NO, using the Griess reagent kit, according to the manufacturer’s instructions. Briefly, microglia cells were seeded in a 12-well plate (1 × 10^6^ cells per well) and preincubated with AE (0.1 μM) or CsA (1 μM) 1 h after the stimulation with LPS (500 ng/mL) for 24 h. Then, in a 96-well plate were mixed 150 μL of cells supernatant, 130 μL of deionized water, and 20 μL of Griess reagent, and the mixture was incubated for 30 min at room temperature. The absorbance values were measured on a spectrophotometer plate reader (548 nm).

### 4.6 Western Blot Analysis

The expression of Nrf2, NF-κβ, p-JNK, JNK, and the iNOS was assessed by Western blot as previously described.^53,54^ In brief, BV2 cells were grown in a 12-well plate and treated as experiments before. Then, cells were centrifuged (1000 rpm and 4 °C for 5 min), rinsed with cold saline buffer and were suspended in 100 μL of ice-cold hypotonic lysis buffer (20 mM Tris-HCl, pH 7.4, 10 mM NaCl, and 3 mM MgCl_2_ containing complete phosphatase/protease inhibitors cocktail). After 15 min of incubation on ice, cells were centrifuged (10 min at 4 °C and 3000 rpm). The supernatant was collected as a cytosolic fraction, and protein concentration was determined by Direct Detect (Millipore). The pellet was suspended in 30 μL of ice-cold nuclear extraction buffer (100 mM Tris, pH 7.4, 2 mM Na_3_VO_4_, 100 mM NaCl, 1% Triton X-100, 1 mM EDTA, 10% glycerol, 1 mM EGTA, 0.1% SDS, 1 mM NaF, 0.5% deoxycholate sodium, and 20 mM Na_4_P_2_O_7_, 1 mM PMSF, and 10× protease inhibitor cocktail) for 30 min and centrifuged (30 min at 14.000*g* and 4 °C). Supernatants were collected as protein nuclear fractions, and their protein concentration was measured using Bradford assay. An amount of 10 μg of nuclear fractions or 15 μg of cytosolic fractions was used for electrophoresis and was resolved on polyacrylamide gels and transferred onto PVDF membrane. Precision Plus Protein Standards Kaleidoscope molecular weight marker was used to determine protein size. Membranes were blocked with 0.5% BSA, and antibody incubation was performed using SNAP i.d. protein detection system. The immunoreactive bands were detected using the SuperSignal West Pico or SuperSignal West Femto and the Diversity GeneSnap software (Syngene). Nrf2 was detected with anti-NF-E2-related factor 2 antibody (1:1000), p-JNK was detected with p-JNK (1:500), JNK with anti JNK (1:1000), iNOS was assessed with anti-iNOS (1:2000) and the NF-κβ with anti-NF-κβ p65 (1:1000). The signal was normalized by that of β-actin (1:2000) for cytosolic samples and by that of laminin B1 (1:1000) for nuclear samples.

### 4.7 Measurement of IL-6 and TNF-α Levels

The amount of IL-6 and TNF-α released by BV2 cells was measured on culture medium supernatants using a magnetic bead-based multiplex immunoassay (Milliplex Map Kit) following the manufacturer's instructions and as described before.^31^ In brief, BV2 cells were seeded in a 12-well plate (1 × 10^6^ cells per well) and treated as described above. The culture medium supernatant was collected and kept at −80 °C until enzyme-linked immunosorbent assay (ELISA) was performed. The median relative fluorescence units were measured using the Luminex 200 with xPONENT software. The samples were analyzed in duplicate, and the concentration was calculated using a standard curve. The minimum detectable concentration is 0.54 pg/mL for IL-6 and 0.41 pg/mL for TNF-α. Culture medium levels below the lower limit of determination were considered as 0 pg/mL for statistical analysis. There was no or negligible cross-reactivity between the antibodies and any other analyte. The intra-assay coefficient of variation of the ELISA kits was <10%. The interassay coefficient of variation of the ELISA kit was <10%.

### 4.8 Microglia and Neuron Coculture System

Neuroblastoma SH-SY5Y cells were grown in the bottom of a 24-well plate, while BV2 microglia cells were grown in culture inserts (pore size 0.4 μm). Through a semipermeable membrane of the insets, the microglia can communicate with the neurons, avoiding direct contact between the two cell lines.^55^ Inserts containing microglia were treated with AE (0.1 μM) or CsA (1 μM) 1 h before LPS treatment (500 ng/mL) for 24 h. Then cellular inserts were removed and MTT assay was performed over SH-SY5Y cells, as described above, to check the cytotoxic effects of activated microglia mediators over neurons.

### 4.9 F- and G-Actin Cytoskeleton Staining and Confocal Microscope Visualization

F- and G-actin levels were analyzed using a confocal microscopy for visualizing morphology and the actin cytoskeleton. After incubation with compounds, for actin labeling, BV2 cells were washed with PBS and fixed with 4% paraformaldehyde. Afterward, cells were permeabilized with PBS-0.1% Triton X-100-2.5% BSA for 5 min and then, for F-actin labeling were incubated with 0.165 μM Oregon Green 514 phalloidin and for G-actin labeling with 0.3 μM Texas Red DNase I for 20 min, followed by PBS washes. Coverslips were mounted in glycerol–PBS and sealed with nail polish and stored at 4 °C.

Images were acquired by using a 40× oil immersion objective in a Nikon Eclipse TE2000-E inverted microscope attached to the C1 laser confocal system (EZC1 V.2.20 software; Nikon Instruments Europe B.V., Netherlands). To excite Oregon Green 514 phalloidin a 488 nm argon laser was used and a 561 nm helium–neon laser for exciting Texas Red DNase I. All laser parameters were adjusted with control cells and remain unchanged for the treated BV2 cells subsequent analysis. Fluorescent images were acquired at a resolution of 512 × 512 pixels separately for each fluorophore and then mixed to avoid interfaces. No interferences of Texas Red in green channel and Oregon Green in red channel were observed.

### 4.10 Statistical Analysis

Results were expressed as mean ± SEM of a minimum of three independent experiments performed by duplicate or triplicate. Comparisons were analyzed using ANOVA statistical analysis followed by *post hoc* Dunnet’s *t*-test. P values <0.05 were considered statistically significant.
